# Biomethanation processes: new insights on the effect of a high H_2_ partial pressure on microbial communities

**DOI:** 10.1186/s13068-020-01776-y

**Published:** 2020-08-10

**Authors:** Lucia Braga Nan, Eric Trably, Gaëlle Santa-Catalina, Nicolas Bernet, Jean-Philippe Delgenès, Renaud Escudié

**Affiliations:** INRAE, Univ Montpellier, LBE, 102 Avenue des Etangs, 11100 Narbonne, France

**Keywords:** Hydrogen, Anaerobic digestion, Biogas upgrading, Power-to-gas, Hydrogenotrophic methanogens, Homoacetogens, *Methanosarcinales*, *Methanosarcina* sp.

## Abstract

**Background:**

Biomethanation is a promising solution to upgrade the CH_4_ content in biogas. This process consists in the injection of H_2_ into an anaerobic digester, using the capacity of indigenous hydrogenotrophic methanogens for converting the injected H_2_ and the CO_2_ generated from the anaerobic digestion process into CH_4_. However, the injection of H_2_ could cause process disturbances by impacting the microbial communities of the anaerobic digester. Better understanding on how the indigenous microbial community can adapt to high H_2_ partial pressures is therefore required.

**Results:**

Seven microbial inocula issued from industrial bioprocesses treating different types of waste were exposed to a high H_2_ partial pressure in semi-continuous reactors. After 12 days of operation, even though both CH_4_ and volatile fatty acids (VFA) were produced as end products, one of them was the main product. Acetate was the most abundant VFA, representing up to 94% of the total VFA production. VFA accumulation strongly anti-correlated with CH_4_ production according to the source of inoculum. Three clusters of inocula were distinguished: (1) inocula leading to CH_4_ production, (2) inocula leading to the production of methane and VFA in a low proportion, and (3) inocula leading to the accumulation of mostly VFA, mainly acetate. Interestingly, VFA accumulation was highly correlated to a low proportion of archaea in the inocula, a higher amount of homoacetogens than hydrogenotrophic methanogens and, the absence or the very low abundance in members from the *Methanosarcinales* order. The best methanogenic performances were obtained when hydrogenotrophic methanogens and *Methanosarcina* sp. co-dominated all along the operation.

**Conclusions:**

New insights on the microbial community response to high H_2_ partial pressure are provided in this work. H_2_ injection in semi-continuous reactors showed a significant impact on microbial communities and their associated metabolic patterns. Hydrogenotrophic methanogens, *Methanobacterium* sp. or *Methanoculleus* sp. were highly selected in the reactors, but the presence of co-dominant *Methanosarcinales* related species were required to produce higher amounts of CH_4_ than VFA.

## Background

Anaerobic digestion (AD) is one of the core technologies contributing to the transition from a fossil fuel-based economy to a more renewable energy-based circular economy [1, 2]. This technology aims to valorize organic residues into an energetic biogas and a digestate that could be used as fertilizer or soil amendment [1]. The energy content of the biogas is proportional to the CH_4_ content. Usually, the AD biogas contains between 40–75% CH_4_ and 25–60% CO_2_, besides other components in minor quantities such as H_2_, N_2_, NH_3_, H_2_S, H_2_O and others trace organic and inorganic components [3]. Because of the presence of CO_2_, the biogas has a lower calorific value than natural gas, i.e. 21.5 MJ/Nm^3^ to 35.8 MJ/Nm^3^, respectively, and therefore, biogas cannot be directly injected into the natural gas grid [4]. The minimal purity of CH_4_ in the natural gas grid must be up to 95%, depending on the countries legislations [5]. Thus, the CH_4_ content in biogas needs to be upgraded prior to be injected into the natural gas grid, used as vehicle fuel or for energy storage.

In order to improve the CH_4_ content in biogas, several purification technologies can be used, and the valorization of the CO_2_ through biological technologies has been recently proposed. In a recent review [5], several biogas upgrading methods were described and discussed. The biological upgrading methods are distinguished as chemoautotrophic or photosynthetic processes. The ex-situ and in-situ biomethanation processes correspond to the chemoautotrophic methods relying on the AD process. In both biomethanation processes, H_2_ is injected into an anaerobic digester in order to upgrade the CH_4_ content by reducing the CO_2_. For the ex-situ biomethanation process, an additional bioreactor, physically separated from the anaerobic digester, is fed with H_2_ and biogas, while for the in-situ biomethanation process, the H_2_ is directly injected into the anaerobic digester [6]. Both processes are based on the capacity of hydrogenotrophic methanogens to use external H_2_ as electron donor for the reduction of CO_2_ into CH_4_ [7], following the reaction:

1 $$4 {\text{ H}}_{ 2} + {\text{ CO}}_{ 2} \to {\text{ CH}}_{ 4} + {\text{ 2 H}}_{ 2} {\text{O }}\Delta {\text{G}}^\circ = \, - 1 30. 7 {\text{ KJ}}/{\text{mol}}$$

H_2_ can have several origins, but to keep the process environment friendly, a renewable energy source should be used [8]. In this way, H_2_ can be generated by water hydrolysis using the energy surplus of wind and solar power plants. However, these renewable power plants produce energy in a fluctuant way, which could lead to imbalance between energy production and the energy demand. Even though H_2_ can also be stored or used as vehicle fuel, the current technologies are today too expensive and technically challenging [9]. Therefore, an alternative is to transform H_2_ into CH_4_, for which storage and transportation are cheaper and, CH_4_ can also be used as vehicle fuel or injected into the gas grid [10, 11]. This concept is named Power-to-Gas (PtG) and fulfils the requirement of linking electrical power and gas-grid networks in an environmentally friendly way.

In this context, both, ex-situ and in-situ biomethanation are suitable processes to be applied to purify biogas produced in AD. However, some challenges need to be addressed prior to develop this technology at industrial scale. One of the limitations concerns the increase of the H_2_ partial pressure within the digester that can alter the AD metabolic equilibrium [7]. The AD process is composed of 4 steps (hydrolysis, acidogenesis, acetogenesis and methanogenesis) which are carried on by different microbial groups that perform these reactions in a very coordinated way. H_2_ is an intermediate during the AD, whose partial pressure, mainly affects the acetogenesis step [12]. During acetogenesis, the volatile fatty acids (VFA) that were formed during the hydrolysis step and the acidogenesis step, are transformed into acetate, H_2_ and CO_2_ [13]. This step is carried out by syntrophic microorganisms, which are thermodynamically constrained by the H_2_ partial pressure, which it must be kept under 10^−4^ atm to allow VFA degradation and methanogenesis [14]. A higher H_2_ partial pressure leads to VFA accumulation in the media, which further inhibits the methanogenic archaeal populations. Nonetheless, the effect of the H_2_ injection over the microbial community is still not very well understood [15]. Mulat et al. [16] and Wahid et al. [17] have reported the inhibition of methane production due to VFA accumulation, caused by H_2_ injection. Moreover, Cazier et al. [18] reported an inhibitory effect due to a high H_2_ partial pressure in the methanogens community, even though the VFA accumulation and pH were not at inhibitory levels. In contrast, in the work of Bassani et al. [19], no hydrolysis inhibition or VFA accumulation was reported after an H_2_ addition and pH increase. Agnesseens et al. [20] detected a transient accumulation of acetate, which was overcome after several H_2_ injections by a microbial adaptation to high H_2_ partial pressure. Therefore, each microbial community configuration can adapt differently to the H_2_ partial pressure. A better understanding of the microbial community characteristics that can improve CH_4_ production under ex-situ and in-situ biomethanation conditions is a key step prior to process optimization [21–23]. This work aims to provide new insights on the influence of the inoculum origin on biomethanation performances, and more particularly on the response of indigenous microbial communities facing high H_2_ partial pressures.

## Results and discussion

### Metabolic patterns production during ex-situ and in-situ biomethanation

Seven microbial inocula coming from different configurations of industrial digestion facilities, and treating diverse types of substrates were inoculated into semi-continuously fed reactors (Table 1). These reactors were fed with (i) H_2_ (ex-situ biomethanation reactors) or (ii) glucose and H_2_ (in-situ biomethanation reactors) in order to compare the effect of H_2_ on the indigenous microbial community during ex situ and in situ biomethanation. Incubations without external CO_2_ addition were chosen in order to simulate a CO_2_-limiting environment.

**Table 1:** Origin of the tested inocula and operational characteristics of the digester of origin

No	Inoculum name	Origin	Type of inoculum	Operational conditions	Treating substrate
1	AnS	Sewage sludge AD	Anaerobic sludge	Anaerobic/continuous/liquid AD/mesophilic	Sewage
2	GS	Paper mill AD	Granular sludge	Anaerobic/UASB/continuous/mesophilic	Paper mill waste
3	BM	Farm AD plant	Liquid fraction (lixiviate)	Anaerobic/discontinuous/dry AD/mesophilic	Manure from bovine livestock
4	MFW1	Territorial AD	Liquid fraction from digestate	Anaerobic/continuous/first stage AD/thermophilic	Poultry slurry and food waste
5	MFW2	Territorial AD	Liquid fraction from digestate	Anaerobic/continuous/second stage AD/mesophilic	Poultry slurry and food waste
6	FW	Territorial AD	Liquid fraction from digestate	Anaerobic/continuous/liquid AD/mesophilic	Food waste
7	AeS	Sewage WWTP	Aerobic sludge	Aerobic/continuous/mesophilic	Waste water

*AD* anaerobic digestion, *UASB* up-flow anaerobic sludge blanket reactor, *WWTP* waste water treatment plant

The consumption of H_2_ started within 24 h after the first H_2_ injection, as consistently reported by Kern et al. [21], Agnesseens et al. [24] and Wahid et al. [17]. Concomitantly, CH_4_ production started 24 h after the first H_2_ injection, and declined along with CO_2_ detection in the head-space (Additional file 1). The sequential injection of H_2_ led to CO_2_ depletion in the head-space of most the reactors between days 4 and 6. At the end of the experiment, a pH increase was observed due to CO_2_ depletion as reported by Luo et al. [9]. Although the ideal pH for methanogens is close to the neutrality, the optimal pH remains variable according to the individual species [7].

The COD mass balance analysis shows that no major metabolites were omitted in this study (Additional file 2), if considering a reasonable variability error of 10% and that at least 10–12% of the electrons contributed to the production of biomass [25–28]. Nonetheless, the carbon conversion was overestimated in some reactors (10–34%), particularly in the ex-situ biomethanation reactors, being attributed to an endogenous methane production [29]. Although, the inocula were stored at ambient temperature ($\cong$ 20 °C) 1 week before use, it was previously reported that an endogenous methanogenic activity was still detected even 10 days after incubation in biochemical methane potential (BMP) tests conditions [30]. In addition, Luo and Angelidaki [8] have attributed the excess of CH_4_ detected in their ex-situ biomethanation reactors to the degradation of the organic matter in the inocula even 10 days after the operation of their reactors have started. Besides, methanogens are considered to have a lower biomass yield than acidogens [27, 31].

**Fig. 1:**
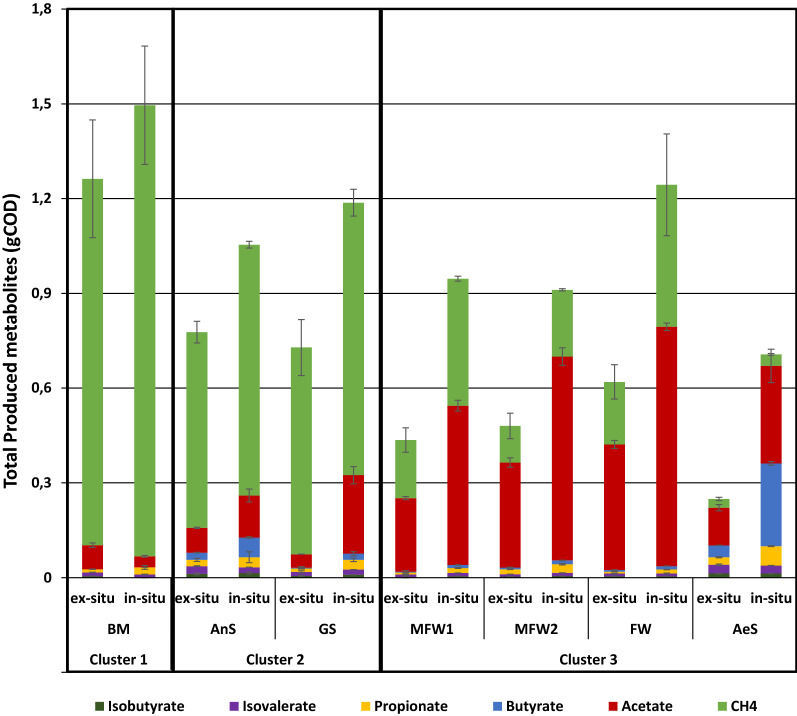
Metabolite production of the ex-situ biomethanation reactors (fed with H_2_ only) and in-situ biomethanation reactors (fed with glucose and H_2_) inoculated with the different inocula and cluster analysis gathering the reactors according to their metabolite production

The average metabolite production (i.e. VFA and methane) of each operating condition is shown in Fig. 1. In all the reactors, after the first H_2_ injection, the H_2_ addition was only made once the total pressure in the reactors was less than 1.2 bar, to maintain an H_2_ partial pressure approximately at 1.0 bar in the head-space (Additional file 1). As some reactors consumed a higher quantity of H_2_, they produced a higher amount of metabolites than other reactors. When the total pressure of the reactors was over 1.2 bar, H_2_ addition was stopped. Liu et al. [32] reported that in batch reactors fed with only H_2_/CO_2_, in a 4:1 proportion, at a H_2_ partial pressure of 0.96 bar, 60% of the H_2_ was used by the hydrogenotrophic methanogens and the other 40% was converted to methane via the association between homoacetogens and acetotrophic methanogens.

A partitioning clustering analysis using the k-means algorithm was performed based on the metabolite production patterns of the reactors (Fig. 1). Three clusters were distinguished according to the source of inoculum and their metabolite production patterns in biomethanation. Cluster 1 corresponds to the reactors (in-situ and ex-situ processes) inoculated with cattle manure leachate from a cattle manure dry anaerobic digestion facility (inoculum BM). These reactors produced almost only methane (94% in average of the total produced metabolites) all along the operation. Cluster 2 is composed of the reactors inoculated with a granular sludge coming from an up-flow anaerobic sludge blanket (UASB) reactor treating paper mill waste (inoculum GS) and the reactors inoculated with a sludge from an anaerobic digester treating aerobic sludge (inoculum AnS). Even though these reactors produced mostly methane ($\cong$ 81% and $\cong$ 78%, respectively, in average from the total produced metabolites), they have also accumulated VFA in their bulk phase, mostly acetate and in lower proportions propionate, butyrate, isobutyrate, and isovalerate. Cluster 3 represents the reactors that accumulated mostly VFA. These reactors were inoculated with the other four inocula used for this experiment (Table 1). One set of reactors was inoculated with the liquid fraction of the first-step reactor from a two-step anaerobic digestion process treating farm waste (principally poultry slurry, food waste and some green waste) (inoculum MFW1), while another set of reactors was inoculated with the liquid fraction of the second-step reactor of the same process (inoculum MFW2). The third set of reactors belonging to cluster 3 was inoculated with the liquid fraction of an anaerobic digester treating food waste (inoculum FW), while the fourth set of reactors belonging to this group was inoculated with an aerobic sludge from a sewage waste water treatment plant (WWTP) (inoculum AeS). A statistically significant difference was confirmed between the clusters regarding their methane production after performing a Kruskal–Wallis test, followed by a Wilcoxon’s test, in which the obtained *p*-values were adjusted with the Bonferroni’s correction method. However, regarding the VFA production only cluster 3 was statistically different from the others (Additional file 3). For the reactors inoculated with MFW1, MFW2 and FW, acetate represented the main accumulated VFA (93%, 94% and 95% in average from the total produced VFA for the reactors inoculated with MFW1, MFW2 and FW, respectively), while the rest of VFA was propionate and traces of butyrate, isobutyrate and isovalerate. Such acetate accumulation is consistent with previous biomethanation performances reported in the literature [9, 24, 33]. On the contrary, the reactors inoculated with AeS produced almost the same proportion of acetate (46%) and butyrate (39%) in average from the total produced VFA. A higher proportion of propionate (10%) was also observed, in comparison with the reactors inoculated with MFW1 (3%), MFW2 (4%) and FW (2%). Some traces of isobutyrate and isovalerate were detected in the reactors inoculated with AeS and fed with glucose and H_2_. In the reactors inoculated with AeS, H_2_ was hardly consumed and the accumulated VFA were likely produced from the organic substrate consumption, leading to the production of other VFA different from acetate. Besides, as these reactors had the lowest H_2_ consumption, H_2_ partial pressure was kept constantly high, likely inhibiting the VFA consumption by the syntrophic microorganisms. Regarding the inoculum AeS, another cluster inside cluster 3 could be detected regarding the accumulation of butyrate and a lower quantity of CH_4_ produced. However, when performing a cluster analysis using the k-means algorithm with *k* = 4, cluster 3 was divided into two groups regarding the amount of total VFA produced: one group containing the ex-situ biomethanation reactors inoculated with MFW1, MFW2, FW and the ex situ and in situ biomethanation reactors inoculated with AeS. While, the other group was formed by the in-situ biomethanation reactors inoculated with MFW1, MFW2 and FW. Nevertheless, the distribution of the groups is more likely related to the amount of COD received by the reactors than with the actual distribution of the metabolite patterns.

For the reactors belonging to cluster 3, a lower quantity of H_2_ was consumed, in comparison with the reactors that produced mostly CH_4_ (clusters 1 and 2). It is likely than in these reactors (cluster 3), when CO_2_ was available, some of it and some of the added H_2_ were transformed into CH_4_ or acetate by the hydrogenotrophic methanogens or homoacetogens, respectively. As a consequence of the CO_2_ depletion, H_2_ started to accumulate in the head-space, likely leading to the accumulation of the VFA coming from the degradation of the remaining organic matter in the inocula, for the ex-situ biomethanation reactors or from glucose degradation in the case of the in-situ biomethanation reactors. According to the observations made by Cazier et al. [18], CO_2_ depletion led to H_2_ and VFA accumulation inducing subsequently the inhibition of syntrophic interactions, and impeding more CO_2_ formation and higher inhibition. In some of the reactors from cluster 3 (inoculated with MFW1, MFW2 and FW) due to H_2_ addition and concomitant CO_2_ depletion, the final pH was over 8.5, which may have contributed to inhibit the methanogens, as already reported in other ex-situ and in-situ biomethanation works [8, 17]. The high accumulation of acetate in these reactors was likely due to the prevalence of homoacetogens over hydrogenotrophic methanogens as more acetate than methane was produced. Agneessens et al. [20] reported that homoacetogens could be more important than hydrogenotrophic methanogens in a low-CO_2_ environment due to their higher resistance to high pH.

The in-situ biomethanation reactors have received a higher amount of substrates (as H_2_ and glucose were provided) than the ex-situ biomethanation reactors (that received only H_2_), likely contributing to a higher production of metabolites as observed in Fig. 1, although the metabolite pattern distribution between in-situ and ex-situ biomethanation reactors was similar.

**Fig. 2:**
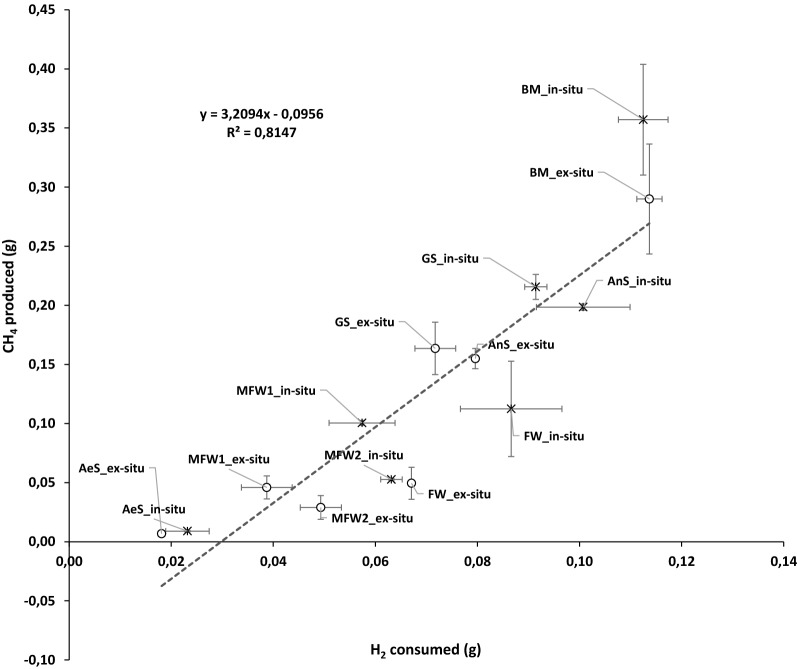
CH_4_ production vs H_2_ consumption

In the reactors belonging to cluster 1 and 2 (reactors inoculated with BM, GS and AnS) that mostly produced CH_4_, lower accumulation of VFA and better H_2_ assimilation were observed. Despite H_2_ consumption have decreased after CO_2_ depletion in the head-space, CH_4_ production positively correlated with H_2_ consumption (*r*^2^ = 0.8147) (Fig. 2), showing that CH_4_ was mostly produced by hydrogenotrophic methanogenesis. In contrast, VFA production did not correlate with H_2_ consumption (data not shown), probably because some of the VFA were consumed to form CH_4_ and, in some reactors, acetate was not exclusively issued from homoacetogenesis. However, acetate production from the reactors in cluster 3, and more particularly the ones inoculated with MFW1, MFW2, FW did positively correlate with H_2_ consumption (*r*^2^ = 0.7615), indicating that at least some of the acetate production came from homoacetogenesis (Additional file 4). The co-occurrence of methanogenesis and homoacetogenesis was already reported by Lay et al [34].

Regarding these results, it is likely that the inocula microbial composition influences the capability of the inocula to use H_2_ in order to produce CH_4_. To our knowledge only the works of Luo and Angelidaki [8] and Bassani et al. [19] have evaluated the influence of the inocula origin during ex-situ and in-situ biomethanation, respectively. Both works have compared the biomethanation potential of a mesophilic inocula under an incubation temperature of 35 °C, to that of a thermophilic inocula under an incubation temperature of 55 °C. These works have reached to the conclusion that a thermophilic operation was more efficient than a mesophilic operation due to their higher methane productivity. However, the growth rate of microorganisms in anaerobic digestion increases linearly between 20 and 60 °C, which improves the efficiency of the process [35, 36]. Therefore, it cannot be determined if the higher methane productivities of the thermophilic inocula were due to the microbial composition or the incubation temperature of the reactors.

### The composition of the indigenous microbial community determines the CH_4_ production and the VFA accumulation after H_2_ addition

The microbial communities of all the reactors were analysed to elucidate the ecological response during ex-situ and in-situ biomethanation. Two samples of each reactor were analysed: the initial inoculum and the final sample for each set of reactors. The effects of H_2_ injection on bacterial and archaeal diversities and dynamics were assessed by 16S rRNA gene sequencing and qPCR analysis.

#### Bacterial community

**Fig. 3:**
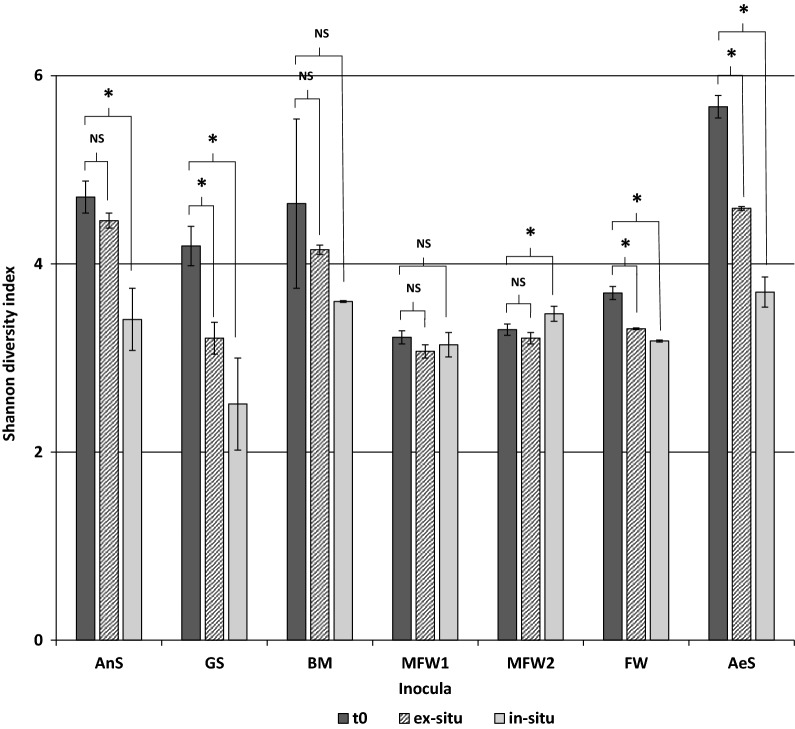
Shannon diversity index calculated for the bacterial community of each inocula. *t*0 indicates the index of the initial inocula. A t-test was performed between the inocula sample and the final sample of the ex-situ or the in-situ biomethanation reactors: statistically significant differences (*p* > 0.05) are shown with an asterisk. *NS* stands for not statistically significant

3872 OTUs were identified and grouped in 98 different classes. Among them, only 17 classes showed a relative abundance in the community higher than 1%. In Fig. 3, the Shannon diversity index of each bacterial community is shown. Most of the inocula have shown a statistically significant decrease of the bacterial community diversity after the H_2_ addition, due to the selective conditions given by the added H_2_. Treu et al. [22] reported a similar decrease in the Shannon entropy index after H_2_ addition in a thermophilic continuous stirred tank reactor (CSTR). Such decrease in diversity of the microbial community was attributed to the high selectivity of H_2_, towards a more specialized community able to use or resist to the added H_2_. Bassani et al. [19] also reported a decrease in alpha diversity of the microbial community when working with thermophilic and mesophilic two-stage biomethanation CSTR treating cattle manure. In the reactors inoculated with farm waste and manure (MFW1 and BM) no difference in the Shannon entropy index was observed between the community of the initial inocula and the communities at the end of the operation, whatever the condition.

**Fig. 4:**
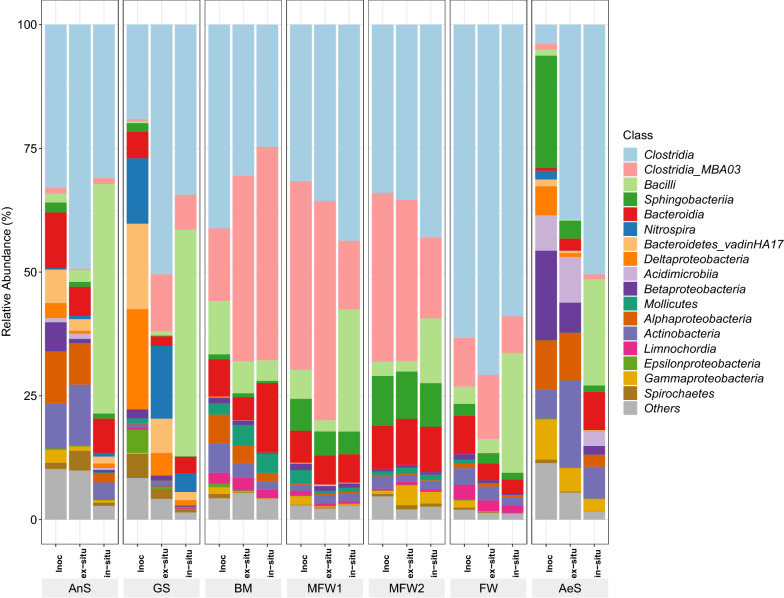
Relative abundance of *Bacteria* classes. The sum of all the OTU with less than one percent abundance is represented under “*Others*”

**Fig. 5:**
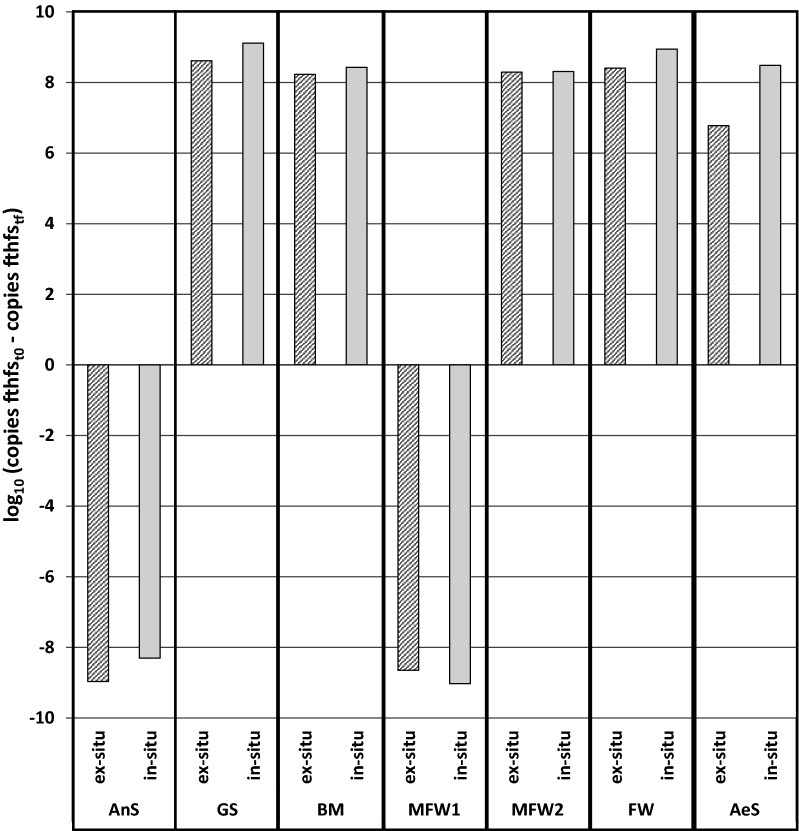
Difference between the initial number of copies of the gene FTHFS per mL of sample (copies FTHFS_t0) and the final number of copies of the FTHFS gene per mL of sample (copies FTHFS_tf) for each reactor

In Fig. 4, the relative abundance of the bacterial community of all reactors is shown. At the beginning of the experiment, the *Clostridia* class was dominant in almost all of the inocula, except in inocula AeS, where the most abundant bacterial classes were related to *Betaproteobacteria* and *Sphingobacteria*. At the end of operation, *Clostridia* class was the most abundant class in all the ex-situ biomethanation reactors and in some in-situ ones, except in the reactors belonging to cluster 2, where *Bacilli* class outcompeted the *Clostridia* class. *Bacilli* was the second most abundant class in all in-situ biomethanation reactors, with exception of the reactors belonging to cluster 1 where *Bacteroidia* class was the second most abundant. *Clostridia*, *Bacilli* and *Bacteroidia* classes are composed of bacteria which are able to hydrolyse polysaccharides, oligosaccharides, rest of plants and manure in complex environments [37]. Some members of *Clostridia* can also perform homoacetogenesis and syntrophic interactions with hydrogenotrophic methanogens [38]. Although *Clostridia* was positively selected in all conditions, in the reactors belonging to cluster 3 where VFA (mostly acetate) accumulated, no well-known homoacetogens were specifically identified among the OTU. This is in contradiction with the qPCR analysis results, which showed an increase in copies of the genes coding for the formyl-tetrahydrofolate synthase (FTHFS) a key enzyme of the Wood–Ljungdahl pathway involved in homoacetogenesis [39]. In Fig. 5 is shown the augmentation or the decrease of the number of copies of the genes coding for FTHFS. A decrease in the FTHFS copy number in all reactors inoculated with AnS (cluster 1) and MFW1 (cluster 3) was detected, although, these sets of reactors had a very different metabolite production profile. Likely the faster depletion of CO_2_ in the reactors inoculated with MFW1 favoured the acetate accumulation [20, 24]. Meanwhile, in the AnS reactors the methane production pathway was favoured because the hydrogenotrophic methanogens have outcompeted the homoacetogens in these reactors. However, the increase in the number of copies of this gene was detected in most of the inocula suggesting a significant presence of homoacetogens (Fig. 5). Homacetogenesis phenotype is widely distributed in the phylogeny, with at least 23 different genera within the *Bacteria* domain and more particularly within the *Firmicutes* phylum that were identified as containing homoacetogenic microorganisms [39]. Since the capacity for carrying a homoacetogenic activity is not always tested as a phylogenetic trait, it is highly probable that already known bacteria may also carry this attribute, but not yet identified [40].

At the beginning of the operation, the most abundant OTU in the reactors inoculated with BM (cluster 1), MFW1 and MFW2 (cluster 3), was affiliated to a cluster named MBA03 at the order level. Although, a BLASTn search against the NCBI database using the 16S rRNA sequence database was performed, no further identification was possible. MBA03 belongs to the *Clostridia* class and was previously found in AD reactors treating cattle manure, silage waste and food waste [41–43]. At the end of the operation, this OTU relative abundance increased in all reactors inoculated with BM. In the ex-situ biomethanation reactors inoculated with MFW1, MFW2 and FW, MBA03 relative abundance was quite stable, it has varied from 38 to 44% in the reactors inoculated with MFW1, from 34% to 33% in the reactors inoculated with MFW2 and from 10 to 13% in the reactors inoculated with FW. In contrast, in the in-situ biomethanation reactors (inoculated with MFW1 and MFW2), the abundance of this OTU decreased from 38 to 14% and from 34 to 16%, respectively. Therefore, this microorganism was likely outcompeted by other members of the *Bacilli* or *Clostridia* classes for glucose.

Some of the OTU belonging to the most abundant groups in the final microbial community were affiliated at a species level, by performing a BLASTn search against the NCBI database using the 16S rRNA sequence database. The identified OTUs related to *Bacilli* were *Amphibacillus xylanus* [44], *Paenibacillus ihumii* [45] and *Vagococcus acidifermentas* [46]. Three of the major OTUs belonging to *Clostridia* were: (i) *Natronincola peptidivorans* [47], (ii) *Proteiniborus ethanoligenes* [48], (iii) *Clostridium isatidis* [49]. All these bacterial species are heterotrophs, mostly peptide- or carbohydrate-consumers, which are able to grow at pH ranging between approximately 6 and 10. Even though the pH of the reactors was measured at the beginning and at the end of the experiment, the pH increase likely started with the CO_2_ depletion as reported elsewhere [17]. Such pH increase probably favoured these species among others, due to their high tolerance to alkaline pH as they could grow even at pH equal to 10.

#### Archaeal community

**Fig. 6:**
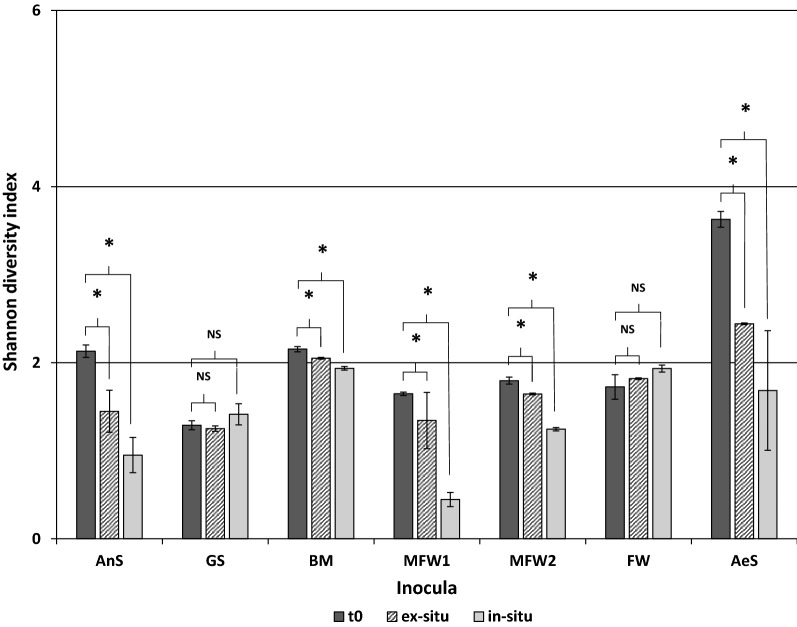
Shannon diversity index calculated for the archaeal microbial community of each inocula. *t*0 indicates the index of the initial inocula. The asterisks indicate that the samples are statistically different (*t* test, *p* < 0.05), while “NS” indicates that not a statistically significant difference exist between samples (*t* test, *p* > 0.05)

461 OTUs belonging to the *Archaea* domain were detected in the sequencing analysis. They were grouped in 20 genera, from which 13 had an abundance higher than 1% in the archaeal community. The Shannon entropy index of the archaeal community in all reactors were smaller than the ones calculated for the bacterial communities, meaning a less diverse community dominated by few distinct OTUs (Fig. 6). A decrease in the diversity was also observed in the archaeal communities in almost all reactors, except in the reactors inoculated with GS and FW where a slightly increase in the diversity of their final microbial communities was noticed. Although, these increase was no statistically significant with regard to the diversity of the inocula (*t* test, *p* > 0.05).

**Fig. 7:**
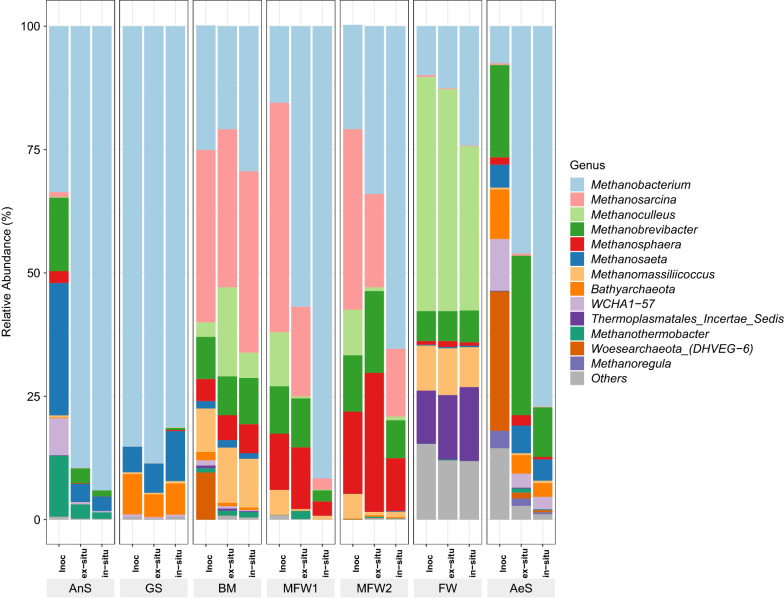
Relative abundance of *Archaea* genera. Only the genus representing more the one percent in at least one of the samples is shown. The term “*Others*” represent the sum of all OTU, which abundances were under 1%

The relative abundance of the archaeal community in all reactors is shown in Fig. 7. In both types of reactors, ex-situ or in-situ ones, the proportion of hydrogenotrophic methanogens increased by the end of the operation, suggesting that a shift in the microbial communities towards a more specialized H_2_-utilizing ones occurred as shown by Agneessens et al. [24]. Meanwhile, the presence of *Methanosarcinales* was detected all along the operation in most of the reactors with a relative abundance greater than 1%, except in the reactors inoculated with FW. The presence and the increase in abundance of *Methanosarcinales* during biomethanation has already been reported [17, 20, 21].

*Methanobacterium* sp. was found to dominate in all the reactors belonging to cluster 2 and in most reactors belonging to clusters 3. Dominance of this genus in biomethanation processes has already been reported [16, 17, 33]. Other genera related to hydrogenotrophic methanogens, in particularly *Methanobrevibacter* sp. and *Methanosphaera* sp. were also found in greater proportion in the ex-situ biomethanation reactors belonging to clusters 3, while in the in-situ biomethanation reactors their proportion diminished with regard to the inocula. Meanwhile, in the reactors belonging to cluster 2, *Methanobacterium* sp. outcompeted the other genera of hydrogenotrophic methanogens (inocula AnS) or were not even present in the initial inocula (inocula GS).

The reactors inoculated with FW were dominated by the genus *Methanoculleus* sp. all along the operation. This genus is composed of hydrogenotrophic methanogens and was also found to dominate the archaeal community during biomethanation [19, 21, 23]. In the reactors belonging to cluster 1, both *Methanobacterium* sp. and *Methanoculleus* sp. increased in proportion in the microbial community by the end of the operation. However, these reactors were dominated by *Methanosarcina* sp. By the end of the operation, the genera *Methanosaeta* sp. and *Methanosarcina* sp. were more abundant or were stable in the reactors belonging to cluster 1 and 2, which produced mostly CH_4_. The presence of these genera probably contributed to avoid acetate accumulation and favoured CH_4_ production.

*Methanosarcina* sp. dominated all along the operation in the all the reactors belonging to cluster 1, although its abundance was reported to decrease because of H_2_ partial pressure inhibition [24]. Interestingly, *Methanosarcina* sp. are able to switch their metabolism from acetate-consuming to H_2_-consuming pathways as a response to the H_2_ partial pressure [50]. Therefore, a high H_2_ availability could be favourable to select hydrogenotrophic methanogens with a lower H_2_ affinity such as *Methanosarcina* sp. [51]. The reactors inoculated with MFW1 and MFW2 (belonging to cluster 3) were dominated by *Methanosarcina* sp. at *t*_0_, although acetate accumulated and the *Methanosarcina* sp. population decreased by the end of the experiment. It is not completely clear why *Methanosarcina* sp. was negatively affected in these reactors. It is possible that the faster CO_2_ depletion due to the H_2_ addition in the reactors inoculated with MFW1 and MFW2 (clusters 3) than in the reactors inoculated with BM (cluster 1) have led to the inhibition of *Methanosarcina* sp. Agneessens et al. [20] and Mulat et al. [16], have attributed such inhibition to low CO_2_ concentration and high H_2_ partial pressure. The loss of *Methanosarcina* sp. was probably the cause of acetate accumulation and low CH_4_ production.

The *Archaea/Bacteria* ratio was calculated for all reactors. The reactors belonging to the clusters 1 and 2, that grouped the reactors that have produced mostly methane (inoculated with AnS, GS, BM) had a significantly higher proportion in *Archaea* at t_0_ (1:21, 1:2.5 and 1:29, respectively), while the other reactors had ratio approximately lower than 1:200 (Table 2). Moreover, in inocula BM (cluster 1) and GS (cluster 2) a higher amount of hydrogenotrophic methanogens (one order of magnitude higher) than homoacetogens was detected (Table 2). Hence, the hydrogenotrophic methanogens were the major hydrogen-utilizing microorganisms in these inocula. Lay et al. [34] reported that the amount of homoacetogens and hydrogenotrophic methanogens in batch co-cultures had an impact in the H_2_ consumption and the favoured pathway to form CH_4_. Such high amount of hydrogenotrophic methanogens might have contributed, at a local scale, to the rapid consumption of H_2_. Moreover, if considering the fact that H_2_ has a low solubility in the liquid media, this could have helped to the decreasing distribution of the solubilized H_2_ from the gas–liquid interphase to the liquid media and therefore, leaving zones in the liquid media where the H_2_ partial pressure was low enough to allow syntrophic interactions [20]. Through these syntrophic microorganisms removing efficiently the VFA, VFA accumulation was avoided and CH_4_ production was enhanced. Nonetheless, the inoculum AnS had a higher amount of homoacetogens than hydrogenotrophic methanogens, although in the same magnitude order. Liu et al. [32] reported that homoacetogens had a lower H_2_ conversion rate than hydrogenotrophic methanogens, meaning that, under same proportions, hydrogenotrophic methanogens will contribute in higher amount to H_2_ consumption than homoacetogens. Since inocula AnS showed the highest amount of *Methanosaeta* sp. among all the inocula, some of the produced methane was likely produced though the homoacetogenic pathway followed by acetotrophic methanogenesis.

**Table 2:** The initial concentration of Bacteria and Archaea (number of copies of the 16S RNA from Bacteria or Archaea gene, respectively, per mL of sample), the initial concentration of homoacetogens (number of copies of the FTHFS gene/mL sample), the initial number of hydrogenotrophic methanogens (number of copies of the 16S archaea gene/mL sample*relative abundance of hydrogenotrophic methanogens in the inocula) and the initial number of acetotrophic methanogens (number of copies of the 16S archaea gene/mL sample*relative abundance of acetotrophic methanogens in the inocula) are shown. As well as, the calculated ratio between the amount of Archaea, respectively, to that of the Bacteria (ratio A:B), the amount of hydrogenotrophic methanogens in relation to the amount of homoacetogens (ratio HM:HA) and to the quantity of acetotrophic methanogens (ratio HM:AM). The sum of the relative abundance of *Methanobacterium* sp., *Methanosarcina* sp., *Methanoculleus* sp., *Methanobrevibacter* sp., *Methanosphaera* sp., *Methanothermobacter* sp. and *Methanospirillum* sp. was used as total relative abundance of hydrogenotrophic methanogens in the inocula, while the sum of the relative abundance of *Methanosaeta* sp. and *Methanosarcina* sp. was used as total relative abundance of acetotrophic methanogens

Inoculum	Total bacteria	Total archaea	Ratio A:B	Total, HA	Total, HM	Total, AM	Ratio HM:HA	Ratio, HM:AM
AnS	1.2 × 10^10^	5.5 × 10^8^	1:21	9.4 × 10^8^	3.6 × 10^8^	1.5 × 10^8^	1:2.6	1:0.4
GS	1.2 × 10^10^	5.0 × 10^9^	1:2.5	2.8 × 10^8^	3.8 × 10^9^	2.3 × 10^8^	1:0.1	1:0.1
BM	3.5 × 10^10^	1.2 × 10^9^	1:29	3.4 × 10^8^	8.3 × 10^8^	3.9 × 10^8^	1:0.41	1:0.5
MFW1	9.8 × 10^10^	4.5 × 10^8^	1:217	1.9 × 10^9^	3.8 × 10^8^	1.9 × 10^8^	1:4.9	1:0.5
MFW2	4.5 × 10^10^	2.7 × 10^8^	1:170	6.3 × 10^8^	2.4 × 10^8^	3.3 × 10^7^	1:2.7	1:0.1
FW	8.0 × 10^10^	9.9 × 10^7^	1:809	1.1 × 10^8^	5.8 × 10^7^	5.0 × 10^5^	1:1.8	1:0.0
AeS	2.9 × 10^10^	8.6 × 10^7^	1:331	1.1 × 10^8^	2.2 × 10^7^	3.9 × 10^6^	1:4.8	1:0.2

Reference: A: archaea, B: bacteria, HA: homoacetogens, HM: hydrogenotrophic methanogens. Inocula: AnS: anaerobic sludge, GS: granular sludge, BM: livestock manure leachate, MFW1: digestate’s liquid fraction from the 1st stage of the anaerobic digestion of farm waste, digestate’s liquid fraction from the 2nd stage of the anaerobic digestion of farm waste, FW: food waste digestate’s liquid fraction, AeS: aerobic sludge

**Fig. 8:**
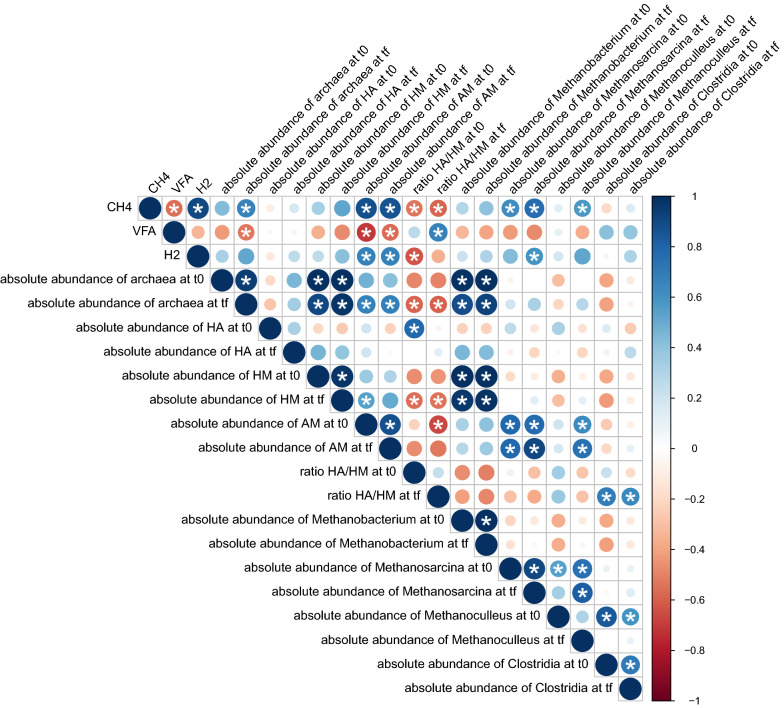
Pearson correlation matrix between the reactors final performances and the microbial communities of the inocula and the final community of the reactors. The asterisk shows that the correlation has a statistical significance (*p* < 0.05). References: H_2_, consumed H_2_ (in gCOD); CH_4_, produced CH_4_ (in gCOD); VFA, produced VFA (in gCOD)

Overall, the final biomethanation performances of the reactors are the outcome of the high selectivity of the H_2_ addition in the initial inocula [22]. Not only the presence of these initial characteristics but also the persistence of them can lead to the production of CH_4_ or the accumulation of VFA, as the possibility of the microbial communities to cope with perturbations is related to the presence of specific tolerant species [52]. A Pearson correlation analysis (Fig. 8) was performed in order to elucidate which features of the inocula could have impacted the final biomethanation performances. To perform the correlation analysis, several features of the inocula were selected. The initial and final amounts of archaea in the inocula were taken into account for this analysis because the reactors grouped in clusters 1 and 2 were inoculated with the inocula that presented higher amounts of archaea in their community. The amount of hydrogenotrophic methanogens and homoacetogens in the inocula likely influenced the predominant metabolite pathway in the microbial community as they are H_2_-consumer microorganisms and their amount in the microbial communities was reported to determine which microorganisms is the principal H_2_ consumer [34]. Hence, their amount and ratio in the inocula and in the final microbial communities of the reactors were taken into account for the analysis. Initial quantity and persistence of the acetotrophic methanogens and the ratio of acetotrophic methanogens to hydrogenotrophic methanogens were also considered. The initial and final amounts of *Methanobacterium* sp., *Methanosarcina* sp., *Methanoculleus* sp. and *Clostridia* were also considered as they were the predominant microorganisms in the microbial communities of the reactors which are able to consume H_2_ to produce CH_4_ or VFA.

From the correlation analysis represented in Fig. 8, the amount of archaea in the inocula did not correlated (negatively or positively) to CH_4_ production or VFA accumulation, although, the persistence of the archaea in the microbial community led to a higher methane production. The initial or final amount of hydrogenotrophic methanogens and homoacetogens did not correlate with H_2_ consumption, nor with CH_4_ production or VFA accumulation, respectively. Interestingly, the ratio homoacetogens to hydrogenotrophic methanogens (HA/HM) in the initial inocula anti-correlated with the CH_4_ production and H_2_ consumption. Meanwhile, the final HA/HM ratio positively correlated to VFA accumulation. Therefore, a higher amount of hydrogenotrophic methanogens than homoacetogens probably favoured H_2_ consumption and methane production. While a higher amount of homoacetogens vs hydrogenotrophic methanogens was correlated with the accumulation of VFA and with the amount of *Clostridia* at the beginning and at the end of the operation. Likely, the homoacetogens present in the reactors belong to the class *Clostridia*. In addition, the amount of acetotrophic methanogens in the initial inocula as well as at the end of operation correlated with methane production and H_2_ consumption and anti-correlated with VFA accumulation, confirming that the persistence of this group of microorganisms is crucial to avoid VFA accumulation.

Even though *Methanobacterium* sp. was one of the most abundant methanogen in all reactors, the increase in its abundance did not correlate to CH_4_ production or H_2_ consumption. In fact, the abundance of *Methanobacterium* sp. increased in all reactors whatever the CH_4_ production. In the reactors from clusters 1 and 2 that have produced mainly CH_4_, *Methanobacterium* sp. was the most (cluster 2) or 2nd most abundant methanogen (in the reactors from cluster 1), although, *Methanosaeta* sp. or *Methanosarcina* sp. were also present, probably contributing to the CH_4_ production, as well. Meanwhile, in the rest of the reactors dominated by *Methanobacterium* sp., an accumulation of VFA was detected. Since CO_2_ was poorly available, this was probably limiting the CH_4_ production by *Methanobacterium* sp. It was previously reported that the CH_4_ production rate of *Methanobacterium* sp. was severely affected by the concentration of CO_2_ in the head-space [24, 53]. The final amount of *Methanoculleus* sp. also correlated with the CH_4_ production likely because, its abundance was highly increased in the inocula of cluster 1 (especially in the ex-situ biomethanation reactors) that mostly produced methane. The reactors grouped in cluster 1 were dominated all along the operation by *Methanosarcina* sp., which was positively correlated, to the CH_4_ production and the H_2_ consumption. *Methanosarcina* sp. is able to stabilize the AD process in adverse conditions such as high H_2_ partial pressure and shift its metabolisms to H_2_ consumption according to the H_2_ partial pressure in the media [50, 54].

## Conclusion

This work has provided new insights on the microbial community response during biomethanation, which are relevant for a practical ex-situ or in-situ biomethanation operation, since the microbial composition of the 7 initial inocula and its maintenance determined the metabolic pathways observed during these processes. According to the results, three main characteristics in the composition of the inocula resulted in better CH_4_ production in biomethanation: (i) an increase or a stability of the *Archaea* proportion from the initial inocula, (ii) a high hydrogenotrophic methanogens/homoacetogens ratio in the inoculum and the persistence or increase of it, and (iii) the initial presence of *Methanosarcinales*, especially *Methanosarcina* sp. and their persistence all along the operation. These new insights would contribute to a more efficient operation of biomethanation reactors.

## Methods

All experiments and physical–chemical analysis were performed at the Bio2E platform [55]

### Inoculum sources

In order to assess how different microbial community structures would respond to H_2_ injections, seven inocula from different origins (Table 1) were exposed to H_2_ injections during 12 days.

Before measuring the concentration of VSS in the UASB inoculum, the granules were broken with immersion blender version WSB33E/K from Waring Commercial^®^ at high speed (20.500 ± 500 rpm) for 10 min. The measurements of TSS and VSS of the inocula were made using the APHA (American Public Health Association) standard methods [56].

### Operational conditions

Schott flasks of 500 mL with a working volume of 200 mL were used and sealed with rubber stopper. The inoculum concentration was 5 gVSS/L. All the inocula were incubated at the same temperature (35°) and agitation speed (370 rpm). The mineral medium was composed of: NH_4_Cl 859 mg/L, KH_2_PO_4_ 323 mg/L, hexa-hydrated MgCl_2_ 194 g/L, di-hydrated CaCl_2_ 97 mg/L, and was supplemented with an oligo-element solution as described in Cazier et al. [18]. Buffer phosphate was also added at a final concentration of 50 mM, at pH 7.5. Two sets of reactors were prepared: (i) feed with H_2_ only (ex-situ biomethanation reactors), and (ii) feed with H_2_ and glucose (in-situ biomethanation reactors). Glucose control was not performed, as in preliminary test carried on in the same conditions, we have detected a pH difference in the media of the reactors fed only with glucose and the ones fed with glucose and H_2_ or H_2_ only (Additional file 5). pH highly influences the amount and the type of produced VFA [57–60], so we could not determine if the difference in the produced VFA would be due to pH effect or to H_2_ injection.

H_2_ injections were carried out with no previous enrichment in hydrogenotrophic methanogens to evaluate the response of the indigenous microbial community to high H_2_ partial pressure. H_2_ injection was made manually by applying a pressure of 1.2 bars. More precisely, H_2_ was injected once a day, only if the total pressure of the vial was lower than 1.2 bars, in order to keep the H_2_ partial pressure around 1 bar, similar to the experiment performed by Liu et al., [32]. The separate injection once a day was done in order to simulate an intermittent H_2_ addition, as if the provided H_2_ was issued from the energy surplus of a wind or solar power plant. Glucose was added every 3 days at final concentration of 0.75 g/L. Thus, the operation was carried on in a semi-continuous regime for 12 days. The experiment duration was based in a previous experiment (Additional file 5), in which after 12 days of operation, the pH has reached almost the value of 9 in some of the reactors and the methane production was nearly stopped. The experiments were performed in duplicates.

### Physical–chemical analysis

Gas pressure and composition were measured twice a day, before and after H_2_ feeding. Liquid samples were taken every day and centrifuged (12,100*g*, 15 min). The supernatant was used to analyse the Volatile Fatty Acid (VFA) concentration while the pellet was kept at − 20 °C for further molecular biology analysis. Gas pressure was manually measured with a manometer Keller LEO 2 (KELLER AG, Winterthur, Switzerland), and gas composition was analysed by gas chromatography using GC Perkin Elmer model Clarus 580, with thermal conductivity detector as described elsewhere by Moscoviz et al. [61]. VFA were analysed by gas chromatography (Perkin Elmer, Clarus 580) coupled with a flame ionization detector as described in Cazier et al. [18]. Glucose concentration of the sample was analysed by YSI 2900D biochemistry analyser, with the corresponding membrane and buffer, according to manufacturer instructions (YSI Inc. Yellow Springs, USA).

### Microbial community analyses

To analyse microbial community composition, Illumina Miseq sequencing and qPCR methods were used. From each reactor, two samples were analysed: the initial and the last-day-of-operation samples. DNA extraction was made with a FastDNA™ SPIN kit in accordance with manufacturer’s instructions (MP biomedicals, LCC, California, USA).

### Bacterial and archaeal community sequencing

The bacteria members were identified by the amplification of the V3–V4 region of the 16S rRNA gene as reported by Carmona et al. [62]. For the identification of archaea members, degenerated primers designed by our laboratory amplifying the V4–V5 region of the 16S rRNA gene were used: 5′-CAGMGCCGCGGKAA-3′ (F504-519) and 5′-CCCGCCWATTCCTTTAAGT-3′ (R910-928). Adapters and bar codes for Miseq sequencing were already included in the primer sets. The PCR mix contained MTP™ Taq DNA Polymerase (Sigma-Aldrich, Inc., Merck, Germany) (0.05 u/µL) with its enzyme buffer, forward and reverse primers (0.5 mM), dNTP (0.2 mM), sample DNA (0.04 to 0.2 ng/µL) and water with a 60µL final volume. The PCR amplification program was the following: 35 cycles of denaturation (95 °C, 1 min), annealing (set at 59 °C, 1 min) and elongation (72 °C, 1 min). At the end of 35 amplification cycles, a final extension step was carried out for 10 min at 72 °C. PCR reactions were carried on in a Mastercycler^®^ thermal cycler (Eppendorf, Hamburg, Germany). All PCR amplifications were verified by 2100 Bioanalyzer (Agilent, Santa Clara, California, USA). The sequencing reaction was carried on in Illumina Miseq sequencer using a 2 × 300 pb paired-end run at the GenoToul platform, Toulouse, France (http://www.genotoul.fr). Reads cleaning, assembly and quality checking was performed in Mothur version 1.39.5. SILVA release 128 was used for alignment and as taxonomic outline [63]. The generated sequencing datasets are registered in the Sequence Read Archive (https://www.ncbi.nlm.nih.gov/sra) under the BioProject accession number PRJNA624130, with SRA accessions SRR11528034 to SRR11528089 for the bacteria-targeted-sequencing dataset and SRR1159475 to 11529530 for the archaea-targeted-sequencing dataset.

### qPCR analysis

Total bacteria, total archaea and the formyltetrahydrofolate synthetase (formate:tetrahydrofolate ligase (ADP-forming), EC 6.3.4.3; FTHFS) gene targeting homoacetogens were analysed by qPCR. All the amplification qPCR programs were performed in a BioRad CFX96 Real-Time Systems C1000 Touch Thermal Cycler (Bio-Rad Laboratories, USA). For total bacteria and total archaea qPCR analysis, primers 338F and 805R and primers 787F and 1059R, respectively, were used [64]. For the bacteria qPCR mix: SsoAdvanced™ Universal Probes Supermix (Bio-rad Laboratories, USA), 338F primer (100 nM), 805R primer (250 nM), TaqMan probe (50 nM), 2 μL of DNA and water was used until a volume of 12.5 μL. The qPCR cycle was the following: 40 cycles of dissociation (95 °C, 7 s) and elongation steps (60 °C, 25 s). The following mix was used for the archaea qPCR reactions: SsoAdvanced™ Universal Probes Supermix (Bio-rad Laboratories, USA), 787F primer and 1059R primer (200 nM), TaqMan probe (50 nM), 5 μL of DNA and water (final volume 25 μL). The qPCR cycle consisted of 40 cycles of denaturation (95 °C, 15 s) and elongation (60 °C, 1 min) (adapted from Braun et al., [64]). For the FTHFS gene, a PCR mix containing: SsoAdvanced™ Universal SYBR^®^ Green Supermix, 500 nM of forward primer and reverse primer (described by Xu et al. [65]), 5 μL of DNA and water for a final volume of 25 μL. The qPCR program consisted in: 2 min at 98 °C, follow by 9 cycles of 45 s at 98 °C and 45 s at 63 °C, each cycle the second set temperature was decreased 1 °C. Finally, 30 cycles 98 °C, 45 s, hybridization (55 °C, 45 s) and elongation (72 °C, 1 min) (adapted from Xu et al. [65]). qPCR results are available in Additional file 1.

### Statistics analysis

All statistical analyses were performed with R software v 3.6.2 using Rstudio v 1.2.1335. The Gap statistic to predict the optimal number of clusters from the used data set (Additional file 1) was calculated with the function “clusGap” of the “cluster” package v 2.0.8. The clusters were calculated using the k-means algorithm from the package “stats” v 3.4.4. The t-test analyses were made using the package “stats” v 3.4.4. The t-test analyses were made using the package “stats” v 3.4.4. The Kruskal–Wallis tests, the Wilcoxon test and the Bonferroni correction method to adjust the p-values for pairwise comparisons, were performed with the “rstarix” v 0.6.0. The Pearson correlation index was calculated with the function “rcorr” from the “Hmsic” v 4.2-0. The representation of the Pearson correlation matrix was made with the “corrplot” package v 0.89 was used. The diversity indexes were calculated with the PhyloSeq package v 1.28.0 [66].

## Supplementary information

**Additional file 1:** The reactors’ performances results and qPCR results.

**Additional file 2: Table S1.** The COD mass balance analysis of the reactors in MS Word document format.

**Additional file 3: Figure S1.** The results of the statistical analysis of the clusters regarding: S1a) the methane production and S1b) the VFA production in PDF format.

**Additional file 4: Figure S2.** The amount of total produced VFA in gCOD vs percentage of consumed H_2_ in MS Word document format.

**Additional file 5:** The results of the preliminary experiment.

## Data Availability

The generated sequencing datasets are registered in the Sequence Read Archive (https://www.ncbi.nlm.nih.gov/sra) under the BioProject accession number PRJNA624130, with SRA accessions SRR11528034 to SRR11528089 for the bacteria-targeted-sequencing dataset and SRR1159475 to 11529530 for the archaea-targeted-sequencing dataset. The reactors’ performances data used for the analysis of the presented results are available in Additional file 1. The qPCR results are also presented in Additional file 1.

## Abbreviations

AD: Anaerobic digestion
AM: Acetotrophic methanogens
BLASTn: Basic local alignment search tool nucleotide
BMP: Biochemical methane potential
CSTR: Continuous stirred tank reactor
FTHFS: Formyl-tetrahydrofolate synthase
HA: Homoacetogens
HM: Hydrogenotrophic methanogens
NCBI: National Center for Biotechnology Information
OTU: Operational Taxonomic Unit
PCR: Polymerase chain reaction
qPCR: Quantitative polymerase chain reaction
SRA: Sequence read archive
UASB: Up-flow anaerobic sludge blanket
VFA: Volatile fatty acids
WWTP: Waste water treatment plant
