# Supplementary material for: Biomethanation processes: new insights on the effect of a high H2 partial pressure on microbial communities
Source: Biotechnol Biofuels. 2020 Aug 10;13:141. doi: 10.1186/s13068-020-01776-y (PMC7419211; doi:10.1186/s13068-020-01776-y)
Supplement: Supplementary file 2 — Additional file 2: Table S1. The COD mass balance analysis of the reactors in MS Word document format. [file 13068_2020_1776_MOESM2_ESM.docx]

**Table S1 –** COD mass balance. Values correspond to the total H_2_ and glucose added to the reactors (H_2__in and glc_in respectively) and the total produced CH_4_, accumulated VFA and the residual H_2_ (H_2__res) in the reactors at the end of the experiment. Residual glucose was 0 for all the reactors at the end of the experiment.

| **Inocula name** | **Fed** | **H_2__in (gCOD)** | **Glc_in (gCOD)** | **CH_4_ (gCOD)** | **VFA (gCOD)** | **H_2_ res (gCOD)** | **COD mass balance (%)** |
| --- | --- | --- | --- | --- | --- | --- | --- |
| **AnS** | H_2_ | 0.86 ± 0.01 | - | 0.62 ± 0.01 | 0.16 ± 0.01 | 0.22 ± 0.02 | 116 ± 3 |
| **AnS** | glc+H_2_ | 0.94 ± 0.02 | 0.40 ± 0.00 | 0.80 ± 0.01 | 0.26 ± 0.03 | 0.14 ± 0.05 | 89 ± 4 |
| **GS** | H_2_ | 0.74 ± 0.02 | - | 0.65 ± 0.09 | 0.07 ± 0.00 | 0.17 ± 0.01 | 121 ± 7 |
| **GS** | glc+H_2_ | 0.81 ± 0.01 | 0.44 ± 0.03 | 0.86 ± 0.04 | 0.24 ± 0.03 | 0.08 ± 0.01 | 101 ± 3 |
| **BM** | H_2_ | 0.95 ± 0.02 | - | 1.16 ± 0.19 | 0.11 ± 0.00 | 0.00 ± 0.00 | 134 ± 23 |
| **BM** | glc+H_2_ | 0.90 ± 0.04 | 0.61 ± 0.02 | 1.43 ± 0.19 | 0.07 ± 0.01 | 0.00 ± 0.00 | 99 ± 8 |
| **MFW1** | H_2_ | 0.55 ± 0.06 | - | 0.18 ± 0.04 | 0.25 ± 0.01 | 0.24 ± 0.02 | 123 ± 4 |
| **MFW1** | glc+H_2_ | 0.70 ± 0.04 | 0.65 ± 0.15 | 0.40 ± 0.01 | 0.54 ± 0.02 | 0.24 ± 0.01 | 89 ± 15 |
| **MFW2** | H_2_ | 0.68 ± 0.04 | - | 0.36 ± 0.04 | 0.12 ± 0.02 | 0.28 ± 0.00 | 113 ± 2 |
| **MFW2** | glc+H_2_ | 0.77 ± 0.01 | 0.54 ± 0.04 | 0.21 ± 0.00 | 0.70 ± 0.03 | 0.26 ± 0.01 | 90 ± 1 |
| **FW** | H_2_ | 0.81 ± 0.00 | - | 0.20 ± 0.05 | 0.44 ± 0.01 | 0.27 ± 0.00 | 110 ± 8 |
| **FW** | glc+H_2_ | 0.96 ± 0.01 | 0.55 ± 0.01 | 0.44 ± 0.16 | 0.79 ± 0.01 | 0.27 ± 0.01 | 100 ± 7 |
| **AeS** | H_2_ | 0.42 ± 0.01 | - | 0.03 ± 0.01 | 0.24 ± 0.02 | 0.27 ± 0.00 | 125 ± 4 |
| **AeS** | glc+H_2_ | 0.45 ± 0.03 | 0.52 ± 0.01 | 0.04 ± 0.00 | 0.72 ± 0.03 | 0.27 ± 0.01 | 98 ± 4 |
