# Supplementary material for: Biomethanation processes: new insights on the effect of a high H2 partial pressure on microbial communities
Source: Biotechnol Biofuels. 2020 Aug 10;13:141. doi: 10.1186/s13068-020-01776-y (PMC7419211; doi:10.1186/s13068-020-01776-y)
Supplement: Supplementary file 3 — Additional file 3: Figure S1. The results of the statistical analysis of the clusters regarding: S1a) the methane production and S1b) the VFA production in PDF format. [file 13068_2020_1776_MOESM3_ESM.pdf]

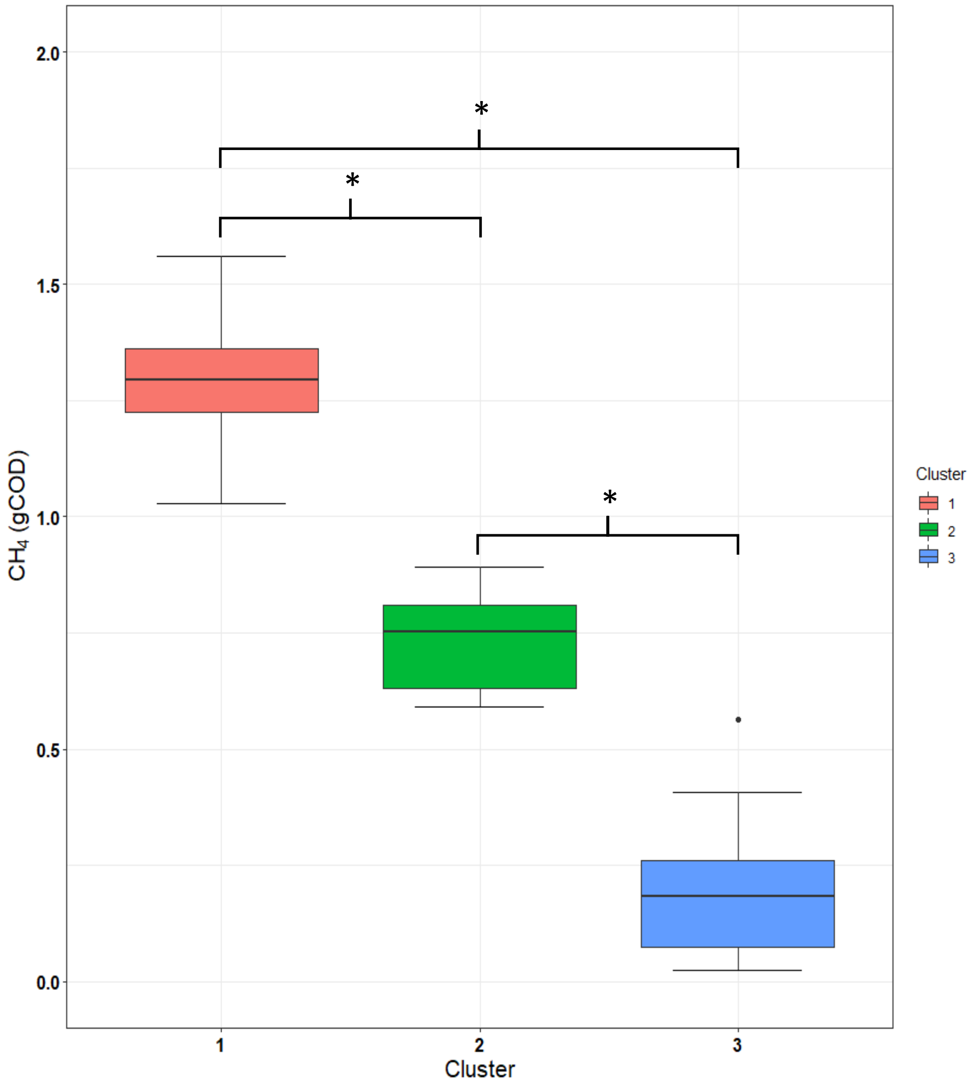

Figure S1a – boxplot showing the distribution of the CH_4 production values in each cluster. Asterisks show a difference statistically significant between clusters (Wilcoxon, $p>0.05$).

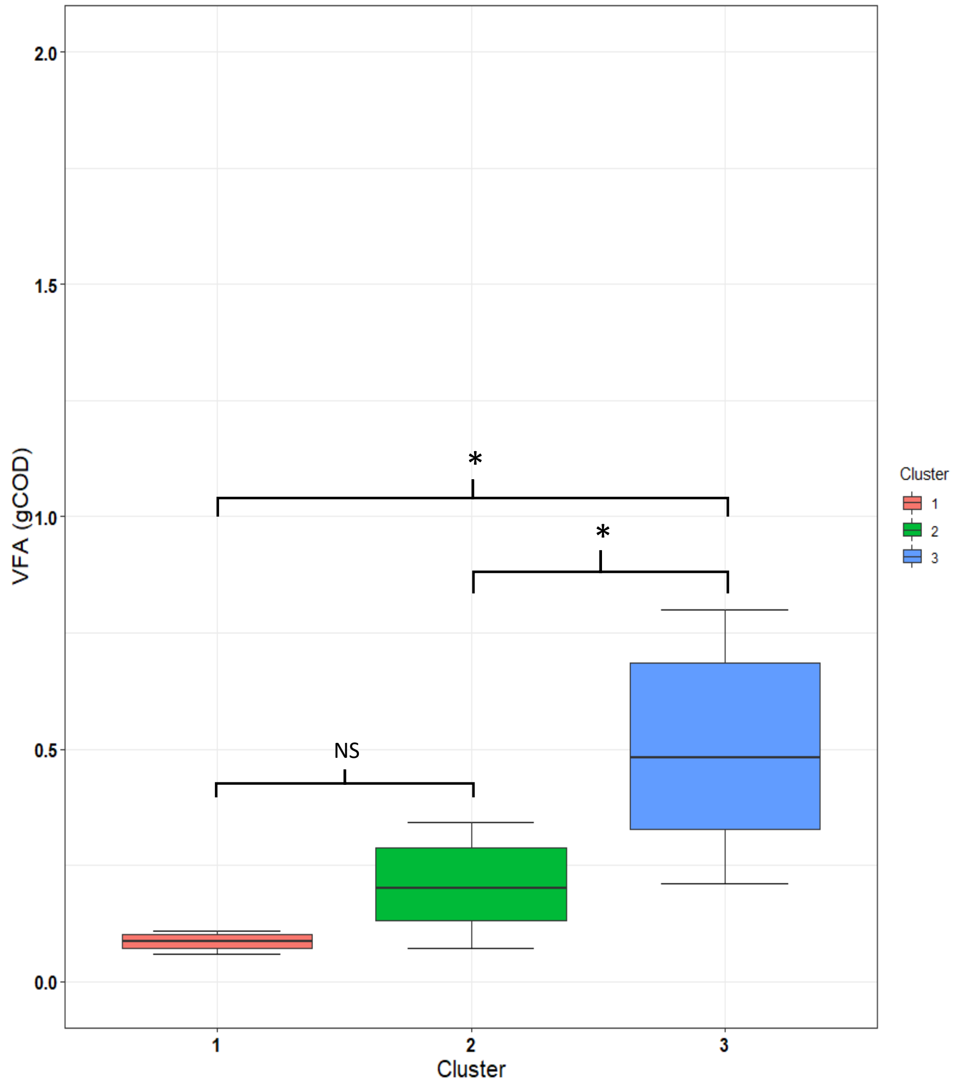

Figure S1b – boxplot showing the distribution of the VFA production values in each cluster. Asterisks show a difference statistically significant between clusters (Wilcoxon, $p>0.05$), "NS" stands for no significance.
