# Supplementary material for: Biomethanation processes: new insights on the effect of a high H2 partial pressure on microbial communities
Source: Biotechnol Biofuels. 2020 Aug 10;13:141. doi: 10.1186/s13068-020-01776-y (PMC7419211; doi:10.1186/s13068-020-01776-y)
Supplement: Supplementary file 4 — Additional file 4: Figure S2. The amount of total produced VFA in gCOD vs percentage of consumed H2 in MS Word document format. [file 13068_2020_1776_MOESM4_ESM.docx]

**Figure S2** – Acetate production in g of the *ex-situ* and *in-situ* biomethanantion reactors inoculated with MFW1, MFW2 and FW vs the g of consumed H_2_ in the same reactors.
